# Surgery-based treatment and prognostic factors in patients with limited-stage small cell lung cancer: a retrospective cohort study

**DOI:** 10.3389/fonc.2026.1807364

**Published:** 2026-05-13

**Authors:** Mingbo Wang, Heng Zhao, Yuefeng Zhang, Huilai Lv, Qin Chu, Yonggang Zhu, Chunyue Gai, Ziqiang Tian

**Affiliations:** 1 Department of Thoracic Surgery, The Fourth Hospital of Hebei Medical University, Shijiazhuang, China; 2 The Fourth Hospital of Shijiazhuang, Shijiazhuang, China

**Keywords:** adjuvant therapy, limited-stage small cell lung cancer, prognosis, surgery, surgery-based treatment

## Abstract

**Introduction:**

Small cell lung cancer (SCLC) is an aggressive malignancy with poor prognosis, and the role of surgery in limited-stage disease remains controversial. This study evaluated the impact of surgery-based treatment for limited-stage SCLC (LS-SCLC) and identified prognostic factors.

**Methods:**

Clinical data of 103 highly selected patients who underwent complete R0 resection and perioperative adjuvant therapy at the Fourth Hospital of Hebei Medical University from 2014 to 2019 were retrospectively analyzed. Disease-free survival (DFS) and overall survival (OS) were assessed by Kaplan-Meier analysis, and independent prognostic factors were determined using multivariable Cox regression.

**Results:**

The cohort included 60 males and 43 females, aged 22–76 years, with a median follow-up of 64 months (IQR: 25–83 months). The median OS was 81 months, and the 5-year OS rate was 58.3%; the median DFS was 50 months, and the 5-year DFS rate was 50.5%. Patients with stage I-IIA disease had significantly better survival than those with stage IIB-IIIB (5-year OS: 69.2% vs. 47.1%, *P* = 0.017; 5-year DFS: 67.3% vs. 33.3%, *P* < 0.001). Multivariable analysis identified advanced pathological Tumor-Node-Metastasis (pTNM) stage (HR = 1.848, 95% CI: 1.156-2.956, *P* = 0.010) as an independent adverse prognostic factor, whereas adjuvant chemotherapy plus thoracic radiotherapy was associated with improved survival. Furthermore, postoperative brain metastasis was a post-treatment progression event associated with markedly poor outcomes (5-year OS: 21.9% vs. 74.6%, *P* < 0.001).

**Conclusions:**

In this highly selected cohort, radical surgery followed by adjuvant chemotherapy and thoracic radiotherapy was associated with improved survival in LS-SCLC, particularly in stage IIB or higher disease. Given the absence of a non-surgical control group, these findings are hypothesis-generating and warrant prospective validation. Postoperative brain metastasis, as a post-treatment progression event, was associated with markedly poor outcomes.

## Introduction

Small cell lung cancer (SCLC) is an aggressive malignancy characterized by rapid progression, poor differentiation, and unfavorable prognosis, representing approximately 13-15% of all lung cancer cases (1). Limited-stage SCLC (LS-SCLC) represents about one-third of all SCLC and refers to tumors confined to one hemithorax, ipsilateral hilar and supraclavicular regions, and the mediastinum, all of which can be encompassed within a single radiation field (2, 3). Although standard chemoradiotherapy has been the cornerstone of treatment, long-term survival remains limited due to high rates of systemic relapse. Consequently, LS-SCLC continues to be associated with unsatisfactory outcomes, highlighting the need for optimized treatment strategies (4).

Traditionally, systemic chemotherapy combined with thoracic radiotherapy has been regarded as the standard of care, based on the notion that SCLC has a strong tendency for early distant metastasis. As a result, surgical resection was historically reserved for only a small subset of patients with very early-stage disease (T1–2N0M0) (5). However, this paradigm has been increasingly challenged. Advances in imaging modalities such as low-dose CT screening, together with the widespread adoption of multidisciplinary team (MDT) management, have facilitated earlier detection and more accurate staging of SCLC. This has led to renewed interest in integrating surgery into a multimodal treatment paradigm across different stages of LS-SCLC (6).

Emerging evidence supports a potential role for surgery in appropriately selected LS-SCLC patients. For example, *Yang* et al. demonstrated that patients with cT1–2N0M0 LS-SCLC who underwent surgery achieved a significantly higher 5-year survival rate than those treated with concurrent chemoradiotherapy (47.6% vs. 29.8%, P < 0.01) (7). Furthermore, contemporary surgical series on locally advanced thoracic malignancies have increasingly highlighted the prognostic importance of nodal stratification and the biological heterogeneity of tumors (8, 9). Large database analyses and multi-institutional studies suggest that integrating surgery into comprehensive treatment regimens, particularly when combined with postoperative adjuvant therapy, can provide survival advantages for biologically favorable subgroups (10). Reflecting this, the International Association for the Study of Lung Cancer (IASLC) has recommended incorporating surgical resection within a multidisciplinary framework, emphasizing the importance of tailoring adjuvant treatment based on pathological staging (11).

However, the optimal postoperative adjuvant strategy across different pathological stages remains unclear, particularly in real-world surgical cohorts. Against this background, we retrospectively analyzed clinical and survival data from a highly selected cohort of LS-SCLC patients who successfully underwent complete R0 resection and received perioperative multimodal adjuvant therapy at our institution. This study aimed to evaluate the survival impact of different postoperative adjuvant strategies and to identify stage-specific prognostic factors in surgically resected LS-SCLC, thereby generating hypotheses and providing real-world evidence to refine patient selection and multimodal treatment strategies.

## Materials and methods

### Study population

We retrospectively reviewed a consecutive series of 138 patients with LS-SCLC who underwent surgical resection in the Department of Thoracic Surgery, Fourth Hospital of Hebei Medical University, between January 1, 2014, and October 31, 2019. The study protocol was approved by the institutional ethics committee (Approval No. 2024K7087).

Inclusion criteria were as follows: (1) histopathological confirmation of SCLC; (2) complete R0 resection; (3) absence of chronic pulmonary diseases (such as chronic obstructive pulmonary disease or pulmonary tuberculosis); and (4) receipt of perioperative adjuvant therapy (chemotherapy and/or radiotherapy). Exclusion criteria included: (1) incomplete perioperative imaging data (including enhanced chest and abdominal CT, enhanced brain MRI, cervical lymph node ultrasonography, bone scintigraphy, and positron emission tomography-computed tomography (PET-CT)); (2) incomplete follow-up information; (3) severe comorbidities involving the heart, lungs, liver, or kidneys, or a history of other malignancies; (4) evidence of contralateral thoracic lymph node involvement or distant organ metastasis prior to treatment. After applying these criteria, a final cohort of 103 patients was included in the analysis.

### Surgical procedures

All surgical procedures were performed by experienced thoracic surgeons. The extent of resection was determined according to tumor location and size, in conjunction with findings from preoperative biopsy or intraoperative frozen-section analysis. Surgical procedures included lobectomy, sleeve lobectomy, combined lobectomy, and pneumonectomy, and were performed via video-assisted thoracoscopic surgery (VATS) or posterolateral thoracotomy.

### Perioperative adjuvant therapy

Neoadjuvant chemotherapy (NACT) was administered to 42 patients with pathologically confirmed SCLC prior to surgery. The regimen consisted of etoposide combined with platinum-based agents, including cisplatin, carboplatin, or nedaplatin, for 1 to 6 cycles (21–28 days per cycle). Etoposide plus cisplatin (EP) was delivered as etoposide 100 mg/m^2^ intravenously on days 1–3 or 1-5, combined with cisplatin 35 mg/m^2^ on days 1-3. Etoposide plus carboplatin (EC) followed the same etoposide schedule, with carboplatin (area under the curve (AUC) = 5) administered on day 2 or the last day of chemotherapy. For the etoposide plus nedaplatin (EN) regimen, etoposide was administered on days 1–3 or 1-5, and nedaplatin was given at 80 mg/m^2^ on days 1-3.

Postoperative chemotherapy (POCT) was administered to all 103 patients within 4–6 weeks after surgery, utilizing the same regimens as those for NACT. A subset of patients also received postoperative thoracic radiotherapy (POTRT). The indication for POTRT was based on postoperative pathological findings, specifically recommended for patients with positive lymph nodes in the ipsilateral hilum, mediastinum, or subcarinal regions. Radiotherapy was delivered via three-dimensional conformal radiotherapy (3D-CRT) or intensity-modulated radiotherapy (IMRT), targeting the bronchial stump, ipsilateral hilum, mediastinum, and subcarinal lymphatic drainage areas. It was initiated concurrently with the second cycle of POCT, at a prescribed dose of 50–60 Gy delivered in 25–30 fractions.

Prophylactic cranial irradiation (PCI) was offered to selected patients without clinical or radiographic evidence of brain metastasis, at a prescribed dose of 25 Gy in 10 fractions. The decision to administer PCI was made by the MDT in consultation with patients and their families.

### Recurrence and metastasis

Local recurrence was defined as the reappearance of tumor at the bronchial stump, ipsilateral hilar lymph nodes, or regional lymph nodes, including mediastinal and supraclavicular nodes (12). Distant metastasis was defined as disease involving the contralateral thorax or distant parenchymal organs such as the contralateral lung, liver, bone, brain, adrenal glands, or supraclavicular/neck lymph nodes (12). Cases exhibiting both local and distant recurrence were documented separately. Lymph node recurrence was established based on radiologic or pathological criteria, including newly detected or enlarged lymph nodes with a short-axis diameter ≥ 1 cm, clusters of ≥ 3 lymph nodes in high-risk drainage regions, progressive enlargement on imaging, shrinkage following antitumor therapy, or high metabolic activity on PET-CT confirmed by pathology (13). Tumor recurrence and staging were evaluated according to the 2009 IASLC lymph node map (13) and the 8th edition of the UICC TNM classification (14).

### Data collection and follow-up

Clinical and pathological data were retrieved from the hospital’s electronic medical record system. Tumor location was classified as central or peripheral, according to established criteria (12). Follow-up began at the date of surgery and continued until October 31, 2024. Data were obtained from electronic medical records, outpatient visits, and telephone interviews. Recorded information included the site and timing of recurrence or metastasis, confirmed by imaging, pathology, or clinical diagnosis. For deceased patients, information on outcomes was obtained from family members.

All survival and prognostic analyses were based on postoperative pathological staging. Specifically, this represents the standard pathological TNM (pTNM) stage for patients who underwent upfront surgery, and the post-neoadjuvant pathological TNM (ypTNM) stage for those who received prior neoadjuvant chemotherapy.

### Outcomes

Overall survival (OS) was defined as the time from surgery to death from primary disease or the last follow-up. Disease-free survival (DFS) was defined as the interval from surgery to the first detection of recurrence, distant metastasis, or last follow-up. Early recurrence was defined as relapse within one year after surgery, while late recurrence was defined as relapse occurring beyond one year (1). Patients who remained alive without evidence of recurrence or metastasis at the last follow-up, or those who were lost to follow-up during the study period, were right-censored at the time of their last clinical contact.

### Statistical analysis

Data analyses were performed using IBM SPSS Statistics version 27.0 (IBM Corp., Armonk, NY, USA). Continuous variables with normal distributions were expressed as means ± standard deviations, and categorical variables were expressed as counts and percentages. Survival curves were generated using the Kaplan-Meier method and compared via the log-rank test.

Variables achieving significance in the univariate analysis (*P* < 0.05), together with variables considered clinically relevant, were entered into the multivariable Cox proportional hazards regression models to identify independent prognostic factors for OS and DFS. To reduce the risk of model overfitting, the number of variables included in the multivariable models was kept limited relative to the number of observed outcome events. Furthermore, to minimize immortal time bias, post-treatment progression events, such as postoperative brain metastasis, were deliberately excluded from the baseline multivariable Cox models. Statistical significance was set at *P* < 0.05.

## Results

### Baseline characteristics

We initially evaluated 138 patients with LS-SCLC who underwent surgical resection. After applying exclusion criteria (13 with incomplete clinical data, 4 without complete R0 resection, 9 with combined SCLC, and 9 who did not receive perioperative adjuvant therapy), 103 patients were included in the final analysis (**Supplementary Figure 1**). The mean cohort age was 57.5 ± 10.1 years (range: 22–76 years), comprising 60 males (58.3%) and 43 females (41.7%).

Upfront surgery was performed in 61 patients (59.2%), whereas 42 patients (40.8%) received NACT prior to resection. Lobectomy was the most common procedure (n = 83, 80.6%), with 20 patients (19.4%) requiring extended resections (combined lobectomy or pneumonectomy). Based on postoperative pathological examination (pTNM), the cohort included 46 patients with stage I disease (including 4 cases of pathological complete response following NACT), 6 with stage IIA, 20 with stage IIB, 30 with stage IIIA, and 1 with stage IIIB.

POCT alone was administered to 82 patients (79.6%), whereas 21 patients (20.4%) received combined POCT and POTRT, which was primarily recommended for patients with postoperative nodal involvement. Baseline demographic and clinical characteristics were generally comparable between the POCT alone and POCT + POTRT groups, with no statistically significant differences observed (**Table 1**). Additionally, PCI was administered to 8 patients (7.8%) (**Supplementary Table 1**).

**Table 1:** Comparison of baseline characteristics between the POCT alone and POCT+POTRT groups.

Characteristics	POCT (n=82)	POCT+POTRT (n=21)	P
Sex (Male/Female)	49/33	11/10	0.541
Smoking history (Yes/No)	33/49	11/10	0.872
Alcohol history (Yes/No)	35/47	9/12	0.766
Age (< 60/≥ 60 years)	42/40	11/10	0.583
Comorbidities (Yes/No)	27/55	8/13	0.655
Tumor laterality (Left/Right)	40/42	11/10	0.933
Tumor distribution (Peripheral/Central)	44/38	11/10	0.917
Surgical approach (Thoracotomy/VATS)	34/48	9/12	0.892
NACT (Yes/No)	33/49	9/12	0.828
Postop chemotherapy (< 4/≥ 4 cycles)	38/44	8/13	0.498
pTNM stage			0.073
I	40	6	
IIA	4	2	
IIB	18	2	
IIIA	19	11	
IIIB	1	0	
PCI (Yes/No)	7/75	1/20	0.564
Resection extent (Lobectomy/Combined)	65/17	18/3	0.098

Data are presented as number of patients. Statistical comparisons were performed using Pearson’s chi-square test or Fisher’s exact test, as appropriate. NACT, neoadjuvant chemotherapy; PCI, prophylactic cranial irradiation; POCT, postoperative chemotherapy; POTRT, postoperative thoracic radiotherapy; pTNM, pathological tumor-node-metastasis; VATS, video-assisted thoracoscopic surgery.

### Survival outcomes

After a median follow-up of 64 months (IQR: 25–83 months), 53 patients developed disease progression, including local recurrence or distant metastasis. Among these, 35 patients had single-organ metastasis, 7 experienced isolated local recurrence, and 11 presented with both local and distant progression.

The median OS for the entire cohort was 81 months, with a 5-year OS rate of 58.3% (**Supplementary Figure 2A**). The median DFS was 50 months, corresponding to a 5-year DFS rate of 50.5% (**Supplementary Figure 2B**).

**Figure 1:**
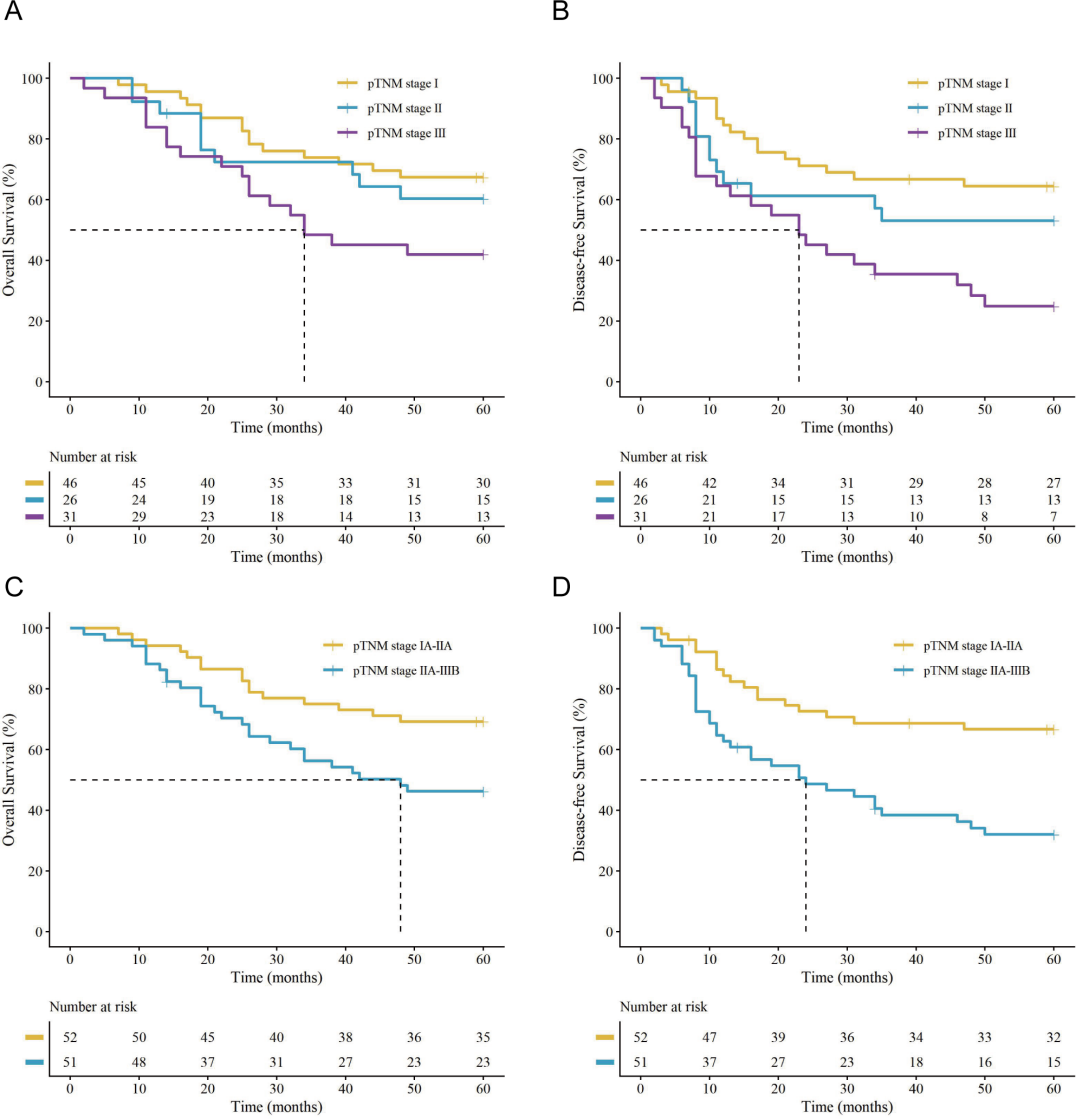
Kaplan-Meier survival curves stratified by pathological TNM (pTNM) stage. **(A)** overall survival (OS) and **(B)** disease-free survival (DFS) for patients in pTNM stages I, II, and III. **(C)** OS and **(D)** DFS comparing patients with early-stage (pTNM I-IIA) versus locally advanced-stage (pTNM II-IIIB) disease. (vertical ‘+’ marks on the curves indicate censored data. The tables below the plots show the number of patients at risk at each time point.).

Survival outcomes differed significantly across pathological stages. The 5-year OS rates were 67.4%, 61.5%, and 41.9% for pTNM stages I, II, and III, respectively (**Figure 1A**), with corresponding DFS curves demonstrating similar significant stage-dependent declines (**Figure 1B**). Patients with early-stage disease (pTNM stage I-IIA) had superior survival compared with those with locally advanced disease (pTNM stage IIB-IIIB), with corresponding 5-year OS rates of 69.2% versus 47.1% (*P* = 0.017) and 5-year DFS rates of 67.3% versus 33.3% (*P* < 0.001) (**Figures 1C, D**).

**Figure 2:**
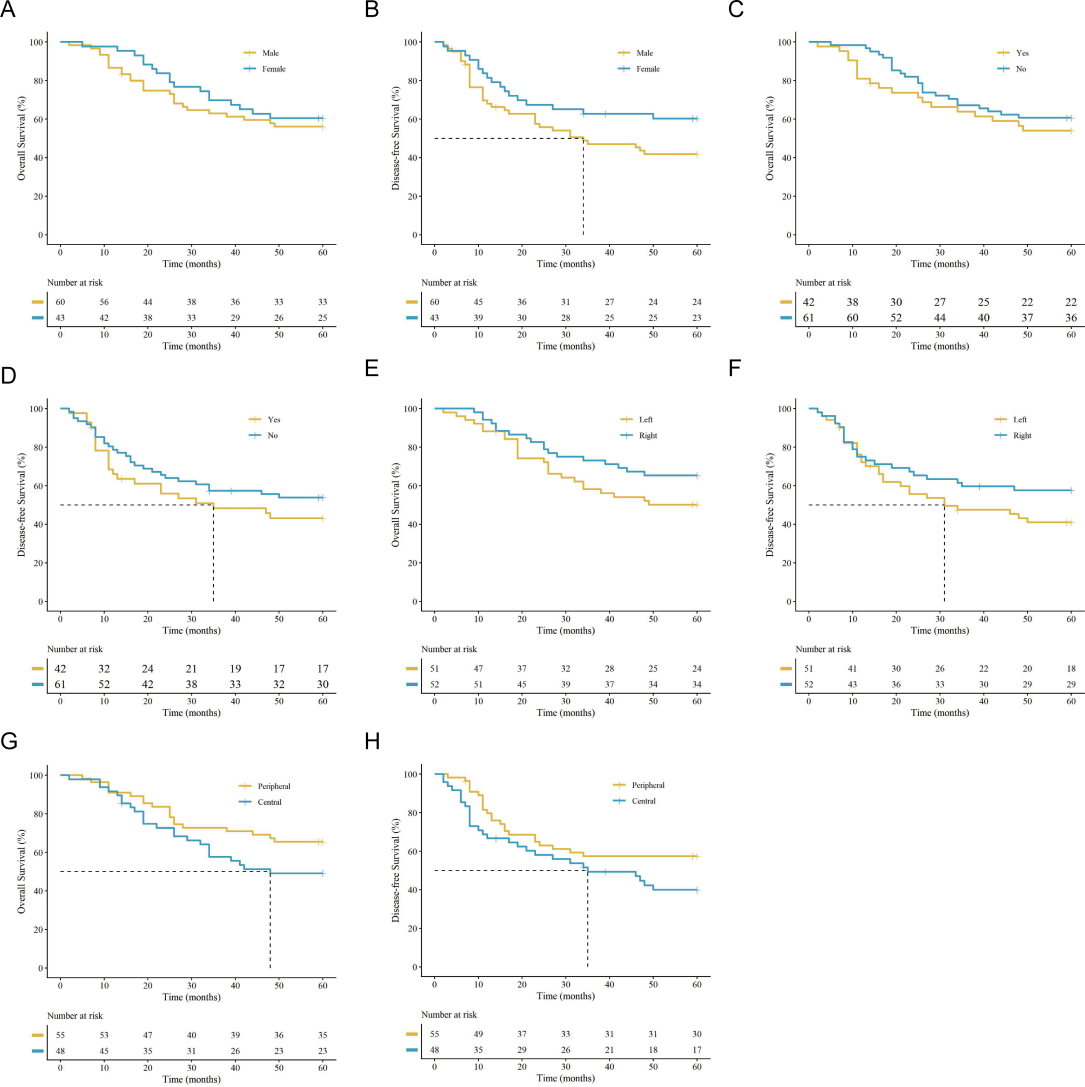
Kaplan-Meier survival curves for univariate analysis of baseline clinical characteristics. OS and DFS stratified by **(A, B)** sex (male vs. female), **(C, D)** smoking history (yes vs. no), **(E, F)** tumor laterality (left vs. right lung), and **(G, H)** tumor distribution (peripheral vs. central). (vertical ‘+’ marks indicate censored data. The tables below the plots show the number of patients at risk.).

**Figure 3:**
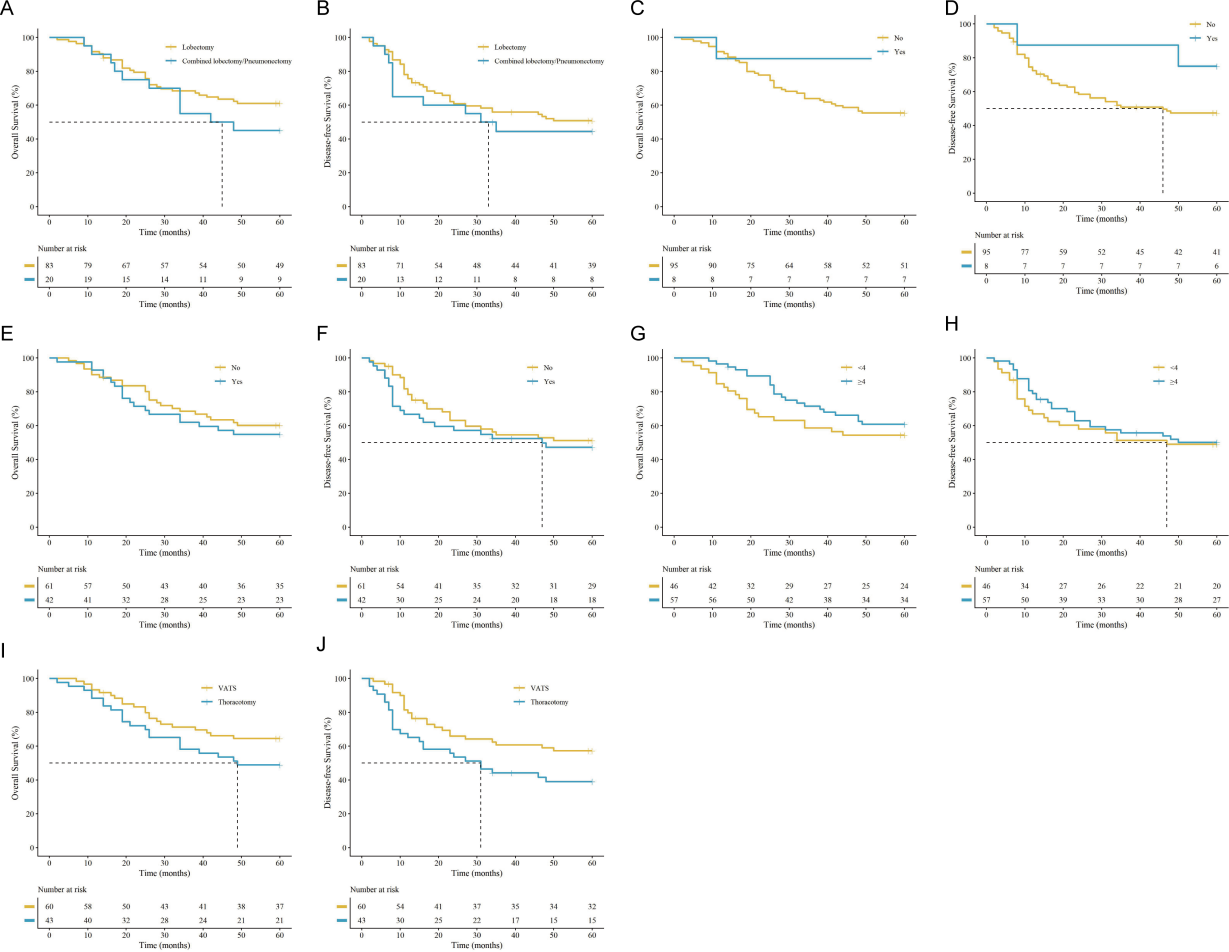
Kaplan-Meier survival curves for univariate analysis of perioperative treatment factors. OS and DFS stratified by **(A, B)** resection extent (lobectomy vs. combined lobectomy/pneumonectomy), **(C, D)** prophylactic cranial irradiation (PCI; no vs. yes), **(E, F)** neoadjuvant chemotherapy (NACT; no vs. yes), **(G, H)** postoperative chemotherapy cycles (< 4 vs. ≥ 4 cycles), and **(I, J)** surgical approach (VATS vs. thoracotomy). (vertical ‘+’ marks indicate censored data. The tables below the plots show the number of patients at risk.).

Univariate analyses evaluated various clinical parameters and revealed no significant differences in OS or DFS according to sex (e.g., 5-year OS for males vs. females: 56.7% vs. 60.5%), smoking history, tumor laterality, tumor distribution, surgical approach, or extent of resection (all *P* > 0.05) (**Figures 2**, **3**). Additionally, OS and DFS were comparable between patients receiving upfront surgery and those receiving NACT (*P* > 0.05). Although patients receiving PCI demonstrated a numerically higher 5-year OS (87.5% vs. 55.8%), this difference was not statistically significant (*P* > 0.05), likely constrained by the small sample size of the PCI subgroup.

### Impact of adjuvant therapy and recurrence patterns

**Figure 4:**
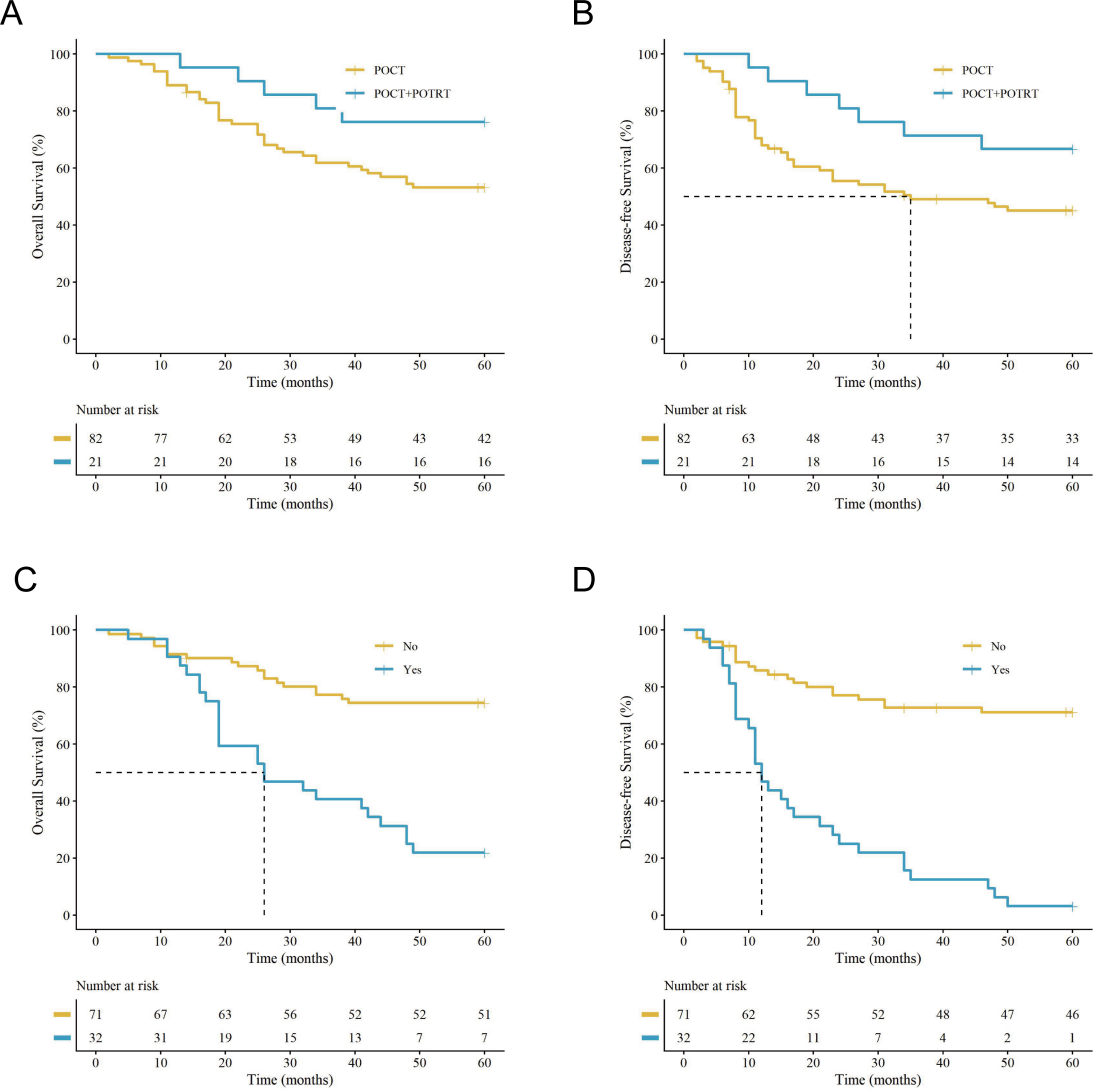
Kaplan-Meier survival curves for univariate analysis of adjuvant therapy modality and postoperative brain metastasis. OS and DFS stratified by **(A, B)** adjuvant treatment modality (POCT alone vs. POCT + POTRT) and **(C, D)** postoperative brain metastasis status (no vs. yes). (note: vertical ‘+’ marks indicate censored data. The tables below the plots show the number of patients at risk.).

Adjuvant treatment modality significantly impacted survival. Combined POCT + POTRT was associated with improved outcomes compared with POCT alone, yielding a 5-year OS of 76.2% versus 53.7% (*P* = 0.041) and a 5-year DFS of 66.7% versus 46.3% (*P* = 0.043) (**Figures 4A, B**).

**Figure 5:**
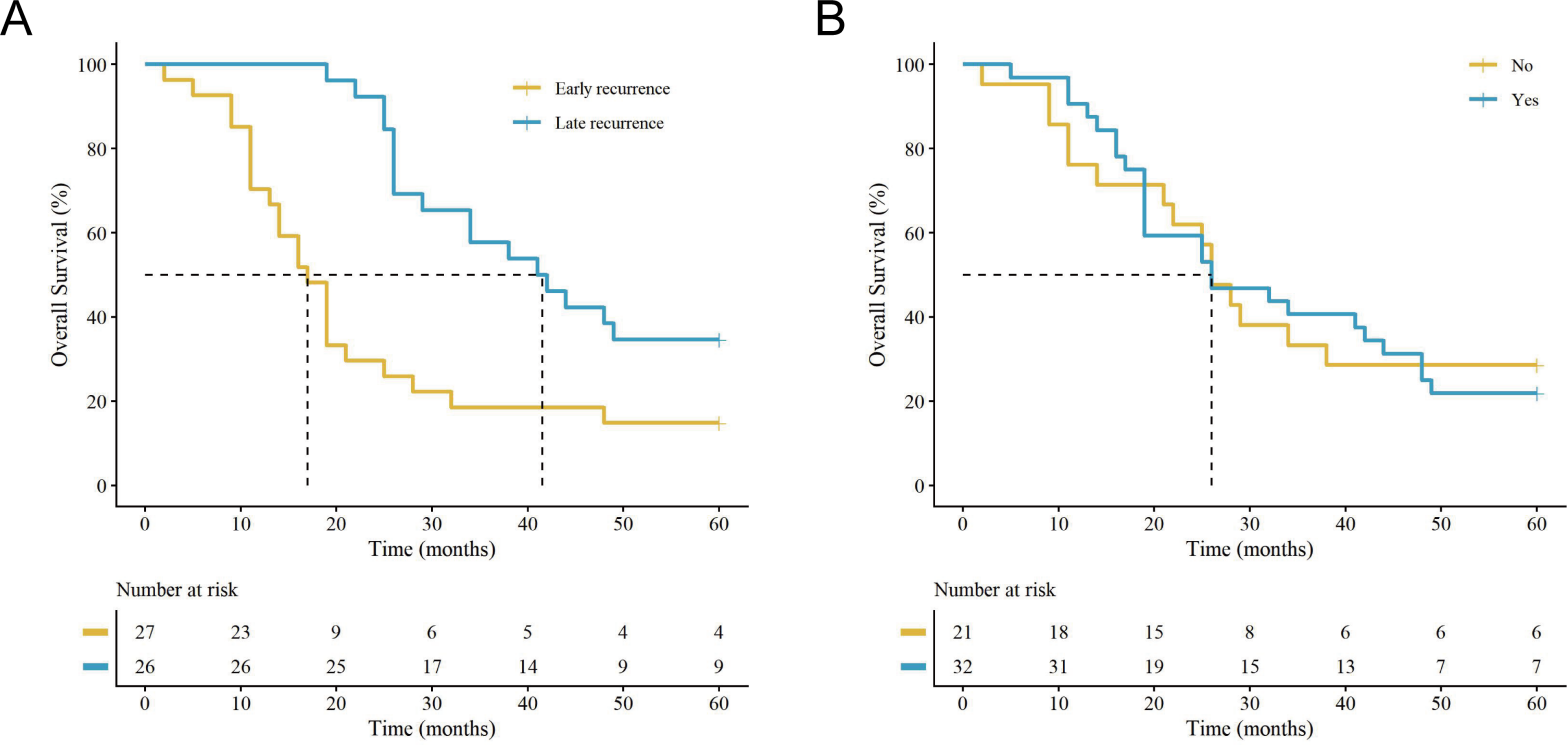
Kaplan-Meier overall survival (OS) curves among the subset of patients who developed disease recurrence (n = 53). OS after recurrence stratified by **(A)** recurrence pattern (early recurrence [≤ 1 year] vs. late recurrence [>1 year]) and **(B)** the presence of brain metastasis among recurrent patients (no vs. yes). (vertical ‘+’ marks indicate censored data. The tables below the plots show the number of patients at risk.).

Postoperative brain metastasis was strongly associated with poor survival outcomes. Patients with brain metastases exhibited a 5-year OS of only 21.9%, compared to 74.6% in those without cranial involvement (*P* < 0.001) (**Figure 4C**). A parallel significant decline was also observed in their DFS curves (**Figure 4D**). However, among the subset of patients who developed disease progression (n = 53), the presence of brain metastasis did not further distinguish OS (21.9% vs. 28.6%, *P* > 0.05) (**Figure 5B**). The timing of recurrence also influenced survival; early recurrence (≤ 1 year postoperatively) was associated with a significantly worse 5-year OS compared to late recurrence (> 1 year) (14.8% vs. 34.6%, *P* < 0.001) (**Figure 5A**).

### Prognostic factor analysis

Univariate log-rank analysis showed that advanced pTNM stage, omission of POTRT, and postoperative brain metastasis were significantly associated with both OS and DFS.

To minimize immortal time bias, post-treatment variables such as postoperative brain metastasis were excluded from baseline multivariable models. Multivariate Cox regression analysis identified combined adjuvant therapy (POCT + POTRT) as an independent protective factor for OS (HR = 0.370, 95% CI: 0.145-0.944, *P* = 0.037) and DFS (HR = 0.283, 95% CI: 0.113-0.710, *P* = 0.007), whereas advanced pTNM stage remained an independent adverse prognostic factor for OS (HR = 1.848, 95% CI: 1.156-2.956, *P* = 0.010) and DFS (HR = 2.084, 95% CI: 1.335-3.251, *P* = 0.001) (**Tables 2**, **3**).

**Table 2:** Univariate and multivariate Cox regression analyses of prognostic factors for overall survival (OS).

Variables	Univariate analysis		Multivariate analysis	
	HR (95% CI)	*P*-value	HR (95% CI)	P
Sex (Male vs. Female)	0.816 (0.442 - 1.503)	0.509	–	–
Smoking history (Yes vs. No)	0.767 (0.420 - 1.401)	0.382	–	–
Tumor laterality (Left vs. Right)	0.623 (0.340 - 1.143)	0.120	–	–
Tumor distribution (central vs. peripheral)	1.640 (0.898 - 2.996)	0.101	–	–
Surgical approach (Thoracotomy vs. VATS)	1.608 (0.884 - 2.925)	0.113	–	–
Brain metastasis (Yes vs. No)	4.318(2.341 - 7.964)	<0.001	–	–
NACT (Yes vs. No)	1.201 (0.658 - 2.192)	0.548	0.803 (0.362 - 1.783)	0.590
POTRT (Yes vs. No)	0.540 (0.301 - 0.980)	0.041	0.370 (0.145 – 0.944)	0.037
Postoperative chemotherapy cycles (≥4 vs. <4)	0.722 (0.397 - 1.314)	0.280	0.530 (0.245 - 1.150)	0.108
pTNM stage	1.493 (1.054 - 2.116)	0.037	1.848 (1.156 - 2.956)	0.010
PCI (Yes vs. No)	0.241 (0.033 - 1.755)	0.124	0.212 (0.028 - 1.584)	0.131
Resection extent (Lobectomy vs. Combined/Pneumonectomy)	1.493 (0.752 - 2.963)	0.244	1.125 (0.486 - 2.609)	0.783

Dash (-) indicates that the variable was not included in the final multivariable Cox proportional hazards regression model. Variables included in the multivariable model were selected based on univariate significance and clinical relevance. Brain metastasis, acting as a post-treatment progression event, was deliberately excluded from the baseline multivariable model to minimize immortal time bias. CI, confidence interval; HR, hazard ratio; NACT, neoadjuvant chemotherapy; OS/DFS, overall survival/disease-free survival; PCI, prophylactic cranial irradiation; POTRT, postoperative thoracic radiotherapy; pTNM, pathological tumor-node-metastasis; VATS, video-assisted thoracoscopic surgery.

**Table 3:** Univariate and multivariate Cox regression analyses of prognostic factors for disease-free survival (DFS).

Variables	Univariate analysis		Multivariate analysis	
	HR (95%CI)	P-Value	HR (95%CI)	P
Sex (Male vs. Female)	0.602 (0.336 - 1.078)	0.080		
Smoking history (Yes vs. No)	0.747(0.430 - 1.298)	0.293		
Tumor laterality (Left vs. Right)	0.673 (0.386 - 1.172)	0.153		
Tumor distribution (Central vs. Peripheral)	1.602 (0.922 - 2.781)	0.087		
Surgical approach (Thoracotomy vs. VATS)	1.697 (0.855 - 3.035)	0.094		
Brain metastasis (Yes vs. No)	6.337 (3.534 - 11.362)	<0.001		
NACT (Yes vs. No)	1.220 (0.701 - 2.125)	0.475	0.902 (0.445 - 1.831)	0.776
POTRT (Yes vs. No)	0.523 (0.213 - 0.981)	0.043	0.283 (0.113 - 0.710)	0.007
Postoperative chemotherapy cycles (≥4 vs. <4)	0.879 (0.506 - 1.526)	0.641	0.657 (0.322 - 1.340)	0.248
pTNM stage	1.694 (1.226 - 2.340)	0.003	2.084 (1.335 - 3.251)	0.001
PCI (Yes vs. No)	0.374 (0.091 - 1.540)	0.151	0.317 (0.074 - 1.364)	0.123
Resection extent (Lobectomy vs. Combined/Pneumonectomy)	1.263 (0.648 - 2.462)	0.487	0.755 (0.345 - 1.652)	0.482

Dash (-) indicates that the variable was not included in the final multivariable Cox proportional hazards regression model. Variables included in the multivariable model were selected based on univariate significance and clinical relevance. Brain metastasis, acting as a post-treatment progression event, was deliberately excluded from the baseline multivariable model to minimize immortal time bias. CI, confidence interval; HR, hazard ratio; NACT, neoadjuvant chemotherapy; OS/DFS, overall survival/disease-free survival; PCI, prophylactic cranial irradiation; POTRT, postoperative thoracic radiotherapy; pTNM, pathological tumor-node-metastasis; VATS, video-assisted thoracoscopic surgery.

## Discussion

SCLC is characterized by early metastasis and a dismal prognosis, and concurrent chemoradiotherapy remains the standard of care for LS-SCLC (1, 15). However, the high incidence of systemic relapse following standard therapy has fueled ongoing debate regarding the role of surgical resection, particularly in patients with more advanced LS-SCLC (15–17). In the present study, we evaluated a highly selected cohort of LS-SCLC patients who underwent radical surgery followed by multimodal adjuvant therapy. Within this cohort, surgery-based multimodal treatment was associated with favorable long-term outcomes, particularly in patients with stage IIB or higher disease. Advanced pTNM stage remained an adverse prognostic factor, whereas postoperative brain metastasis, as a post-treatment progression event, was associated with markedly poor survival.

Previous reports indicate that the 5-year survival rate for LS-SCLC treated with concurrent chemoradiotherapy ranges between 19% and 37% (18, 19). Retrospective surgical series have reported more favorable outcomes in appropriately selected patients. *Che* et al. analyzed 4,780 patients with stage I–III LS-SCLC from the SEER database and found that lobectomy was associated with significant survival benefits (20). Similarly, *Zhong* et al. reported markedly better median OS (79 vs. 23 months, *P* < 0.0001) and median DFS (73 vs. 10.5 months, *P* < 0.0001) in surgically treated patients compared to those receiving chemoradiotherapy (21). In our study, the median OS and DFS were 81 and 50 months, respectively, with corresponding 5-year OS and DFS rates of 58.3% and 50.5%. Even among patients with stage III disease, the 5-year OS and DFS reached 41.9% and 25.8%, respectively. Taken together, these findings suggest that surgery-based multimodal therapy may be associated with favorable survival in selected LS-SCLC patients. However, given the absence of a direct non-surgical control group in our cohort, comparisons with historical chemoradiotherapy outcomes should be interpreted cautiously, and the present findings should be regarded as hypothesis-generating.

While current National Comprehensive Cancer Network (NCCN) guidelines restrict surgery to early-stage (cT1–2N0M0) LS-SCLC (5), accumulating evidence suggests that selected patients with more advanced locoregional disease may also derive benefit from surgery when managed within a multimodal framework. Several population-based studies and institutional series have reported favorable outcomes in selected patients with stage IIB–IIIB disease treated with surgery combined with adjuvant chemotherapy and/or radiotherapy (4, 22–25). In our cohort, patients with stage IIB or higher disease achieved a median OS of 48 months, with 5-year OS and DFS rates of 47.1% and 33.3%, respectively. Although these findings should be interpreted cautiously given the retrospective design, lack of a non-surgical control group, and inherent selection bias, they suggest that the role of surgery in selected stage IIB–III LS-SCLC warrants further investigation within a multimodal treatment strategy.

pTNM stage emerged as an independent prognostic factor in our analysis. *Zeng* et al. reported that the risk of death was significantly higher in stage II and III LS-SCLC compared with stage I disease, confirming pTNM stage as an independent predictor of survival (26). Similarly, *Wei* et al. demonstrated, using SEER-based propensity score analysis, that earlier T and N stages were strongly associated with better survival (27). These observations in LS-SCLC align with contemporary surgical series evaluating locally advanced thoracic malignancies. Recent cohorts of stage III and T4 non- SCLC have similarly highlighted the prognostic significance of meticulous nodal stratification and tumor biological heterogeneity (8, 9). Furthermore, studies focusing on extended anatomical resections have demonstrated the efficacy of multimodal therapy, confirming the independent survival benefit of adjuvant chemotherapy in advanced-stage disease (8, 9). Consistent with these broader thoracic oncology principles, careful patient selection, precise nodal evaluation, and the integration of adjuvant systemic therapies are critical for improving long-term outcomes, independent of the specific histological subtype.

The optimal postoperative adjuvant treatment strategy for LS-SCLC remains controversial. In our clinical practice, POTRT was primarily recommended for patients with pathological N1 or N2 nodal involvement, as determined by MDT consensus. Although only 20.4% of the cohort (n = 21) received POTRT, measured baseline characteristics were generally comparable between the POCT alone and POCT + POTRT groups, with no statistically significant between-group differences observed in **Table 1**. Nevertheless, because treatment allocation was not randomized and was partly guided by postoperative nodal status, residual confounding by indication cannot be excluded. Furthermore, although the difference was not statistically significant (P = 0.073), a higher proportion of patients with stage III disease received POCT+POTRT, indicating a potential imbalance in disease severity between groups.​ Within these limitations, patients receiving combined POCT + POTRT achieved better OS and DFS than those treated with POCT alone, suggesting that thoracic radiotherapy may provide additional benefit in selected surgically treated patients. However, the number of chemotherapy cycles was not associated with improved outcomes, indicating that treatment efficacy may depend more on tumor stage and radiotherapy integration than on chemotherapy intensity alone.

The role of PCI in surgically treated LS-SCLC remains poorly defined. While some meta-analyses indicate survival benefits (28), other studies show limited efficacy in early-stage patients with a low baseline risk of brain metastasis (29). Additionally, the protective effect of hippocampal-sparing techniques during PCI remains controversial (30). The administration of PCI in our cohort was notably low (7.8%). This reflects the ongoing debate regarding its absolute survival benefit in completely resected early-stage disease. Furthermore, patients frequently decline whole-brain irradiation due to well-documented concerns regarding subsequent neurocognitive decline. Given the small number of patients who received PCI in our cohort, no definitive conclusions can be drawn, and further large-scale studies are required to clarify the precise indications for PCI in this surgical population.

The value of NACT is also debated. *Miao* et al. found that NACT did not significantly improve survival in LS-SCLC, regardless of lymph node status (31). Similarly, in our study, survival outcomes did not differ significantly between patients receiving NACT and those undergoing upfront surgery. This may be partly explained by the high proportion of advanced-stage patients in the NACT group and the limited downstaging effect observed. Nonetheless, emerging studies suggest that combining NACT with immunotherapy may enhance response rates and expand surgical eligibility (32). With immunotherapy now established in extensive-stage SCLC, its integration into treatment for LS-SCLC represents a promising area of research.

A notable phenomenon in our cohort was the discrepancy between preoperative clinical staging (cTNM) and pTNM, with several patients being clinically downstaged after surgery. This primarily stems from the inherent limitations of preoperative imaging modalities such as enhanced CT or PET-CT. Tumors accompanied by obstructive pneumonia or reactive lymph node hyperplasia frequently cause false-positive nodal enlargement, leading to an overestimated clinical stage (33). This discrepancy underscores the critical diagnostic value of surgical intervention in establishing a definitive pathological stage, which is essential for determining reliable prognosis and tailoring adjuvant therapy.

Our study has several limitations. Most notably, as a single-center retrospective analysis, the strict inclusion of consecutive patients who successfully achieved complete R0 resection and subsequently completed perioperative adjuvant therapy introduces substantial selection bias. Because the primary objective of this study was to evaluate the efficacy of a surgery-based multimodal treatment paradigm, patients who did not receive adjuvant therapy were excluded. Importantly, patients who were deemed unresectable or medically ineligible for surgery were not captured in this study, further emphasizing the highly selected nature of the cohort. To provide clinical context, out of approximately 15,000 lung cancer surgeries performed at our center during the 5-year study period, only 138 patients were pathologically confirmed to have SCLC, and nearly half of them proceeded to surgery without a definitive preoperative pathological diagnosis. Consequently, our cohort inherently represents a biologically favorable subgroup with better performance status. This restricts the generalizability of our findings to the broader LS-SCLC population, particularly those who are deemed unresectable or are unable to tolerate intensive multimodal therapy. Furthermore, postoperative brain metastasis was excluded from the multivariable model to avoid immortal time bias. In addition, given the retrospective design, limited sample size, and the number of observed outcome events, the multivariable Cox regression results should be interpreted with caution (34). Notably, the proportional hazards assumption for the Cox models was not formally tested, which is a methodological limitation.​ Although the models were kept relatively parsimonious and post-treatment events were excluded from the baseline models, the possibility of residual model instability cannot be completely excluded. Finally, the non-randomized allocation of POTRT, which was primarily guided by postoperative nodal status (N1/N2 disease), may introduce confounding by indication. Though no statistically significant difference in the distribution of the overall pTNM stage between groups, we agree that a numerical imbalance exists (e.g., a higher proportion of stage III patients in the POCT+POTRT group) and that this could serve as a source of residual confounding.

In summary, surgery-based multimodal therapy was associated with improved survival in LS-SCLC, particularly in patients with stage IIB or higher disease. Neoadjuvant chemotherapy alone did not confer a survival advantage. However, given the absence of a non-surgical control group, these findings should be interpreted as hypothesis-generating and require validation in prospective studies.

## Data Availability

The original contributions presented in the study are included in the article/**Supplementary Material**. Further inquiries can be directed to the corresponding author.

## Supplementary material

Supplementary Figure 1 Flowchart of patient selection and inclusion for the study. This flowchart details the selection process, excluding patients without complete R0 resection or adjuvant therapy, resulting in a final cohort of 103 limited-stage small cell lung cancer (LS-SCLC) patients.

Supplementary Figure 2 Kaplan-Meier survival curves for the entire study cohort. **(A)** OS and **(B)** DFS for all 103 patients with LS-SCLC. (Vertical ‘+’ marks indicate censored data. The tables below the plots show the number of patients at risk.).
