# Supplementary figures and images for: Surgery-based treatment and prognostic factors in patients with limited-stage small cell lung cancer: a retrospective cohort study
Source: Front Oncol. 2026 May 13;16:1807364. doi: 10.3389/fonc.2026.1807364 (PMC13212093; doi:10.3389/fonc.2026.1807364)

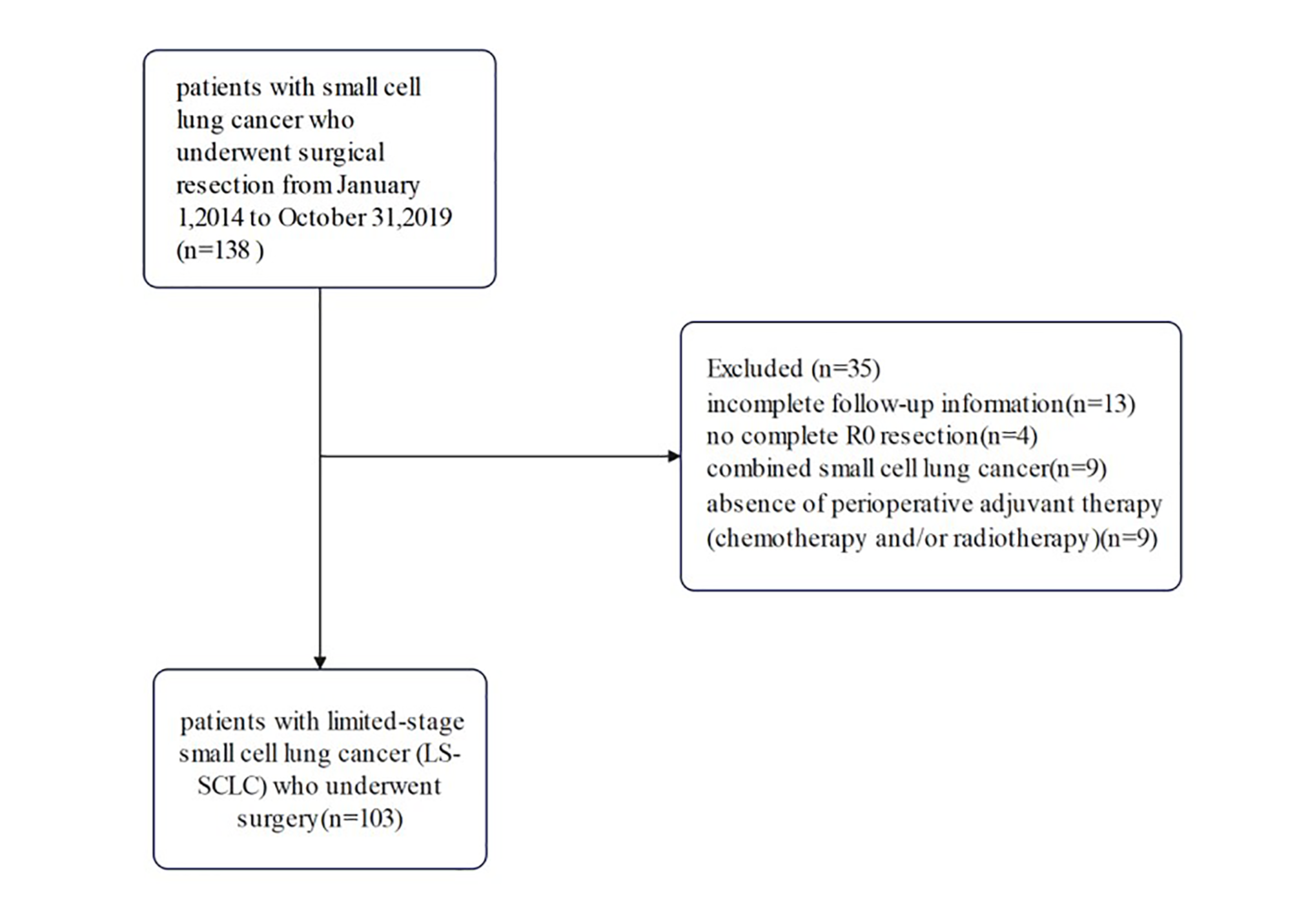

Supplement: Supplementary Figure 1 — Flowchart of patient selection and inclusion for the study. This flowchart details the selection process, excluding patients without complete R0 resection or adjuvant therapy, resulting in a final cohort of 103 limited-stage small cell lung cancer (LS-SCLC) patients. [file Image1.tif]

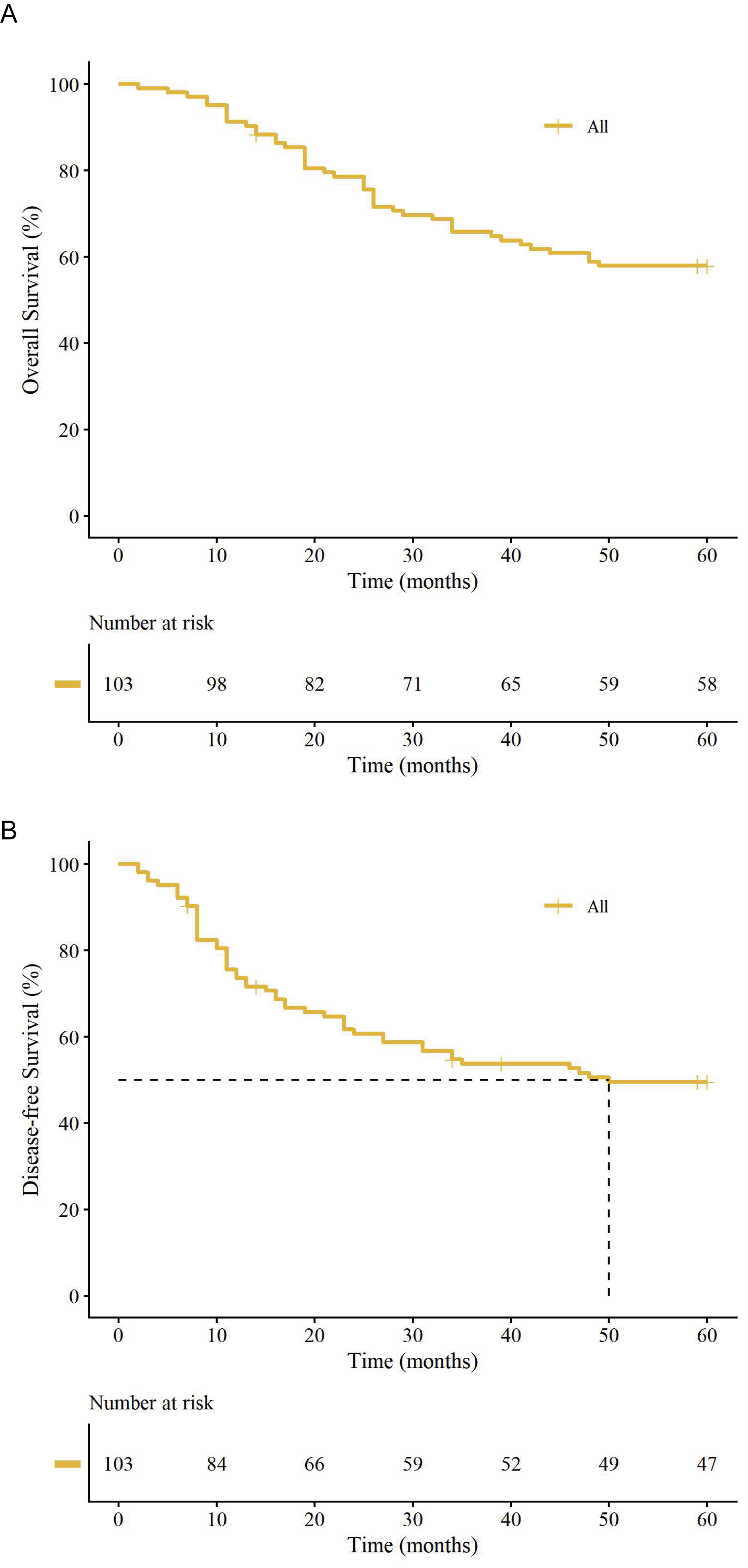

Supplement: Supplementary Figure 2 — Kaplan-Meier survival curves for the entire study cohort. (A) OS and (B) DFS for all 103 patients with LS-SCLC. (Vertical ‘+’ marks indicate censored data. The tables below the plots show the number of patients at risk.). [file Image2.tif]
