# Supplementary material for: Surgery-based treatment and prognostic factors in patients with limited-stage small cell lung cancer: a retrospective cohort study
Source: Front Oncol. 2026 May 13;16:1807364. doi: 10.3389/fonc.2026.1807364 (PMC13212093; doi:10.3389/fonc.2026.1807364)
Supplement: Supplementary file 3 [file Table1.docx]

| **Supplementary Table 1. Baseline clinical and recurrence characteristics of all 103 patients with limited-stage small cell lung cancer** | |
| --- | --- |
| **Characteristics** | **Total (n = 103)** |
| **Age (years),** mean ± SD (range) | 57.46 ± 10.13 (22-76) |
| **Sex (Male / Female)**, n (%) | 60 (58.3) / 43 (41.7) |
| **Smoking history (Yes / No)**, n (%) | 42 (40.8) / 61 (59.2) |
| **Alcohol history (Yes / No),** n (%) | 44 (42.8) / 59 (57.2) |
| **Comorbidities (Yes / No),** n (%) | 35 (34.0) / 68 (66.0) |
| **Surgical approach (Thoracotomy / VATS)**, n (%) | 43 (41.7) / 60 (58.3) |
| **Tumor distribution (Peripheral / Central)**, n (%) | 55 (53.4) / 48 (46.6) |
| **Tumor laterality (Left / Right)**, n (%) | 51 (49.5) / 52 (50.5) |
| **Resection extent (Lobectomy / Combined)**, n (%) | 83 (80.6) / 20 (19.4) |
| **NACT (Yes / No)**, n (%) | 42 (40.8) / 61 (59.2) |
| **POTRT (Yes / No)**, n (%) | 21 (20.4) / 82 (79.6) |
| **PCI (Yes / No)**, n (%) | 8 (7.8) / 95 (92.2) |
| **Postop chemotherapy (< 4 / ≥ 4 cycles)**, n (%) | 46 (44.7) / 57 (55.3) |
| **pTNM stage**, n (%) |  |
| I | 46 (44.7) |
| IIA | 6 (5.8) |
| IIB | 20 (19.4) |
| IIIA | 30 (29.2) |
| IIIB | 1 (1.0) |
| **Recurrence and Metastatic Patterns**, n (%) |  |
| Brain metastasis only (Yes / No) | 21 (20.4) / 82 (79.6) |
| Bone metastasis only (Yes / No) | 8 (7.8) / 95 (92.2) |
| Liver metastasis only (Yes / No) | 4 (3.9) / **99** (96.1) |
| Adrenal metastasis only (Yes / No) | 1 (1.0) / 102 (99.0) |
| Supraclavicular/neck LN metastasis only (Yes / No) | 1 (1.0) / 102 (99.0) |
| Local recurrence only (Yes / No) | 8 (7**.8**) / 95 (**92.2**) |
| **Number of recurrence/metastatic sites**, n (%) |  |
| 0 | 50 (48.5) |
| 1 | 42 (40.8) |
| ≥ 2 | 11 (10.7) |

**Abbreviations:** LN, lymph node; NACT, neoadjuvant chemotherapy; PCI, prophylactic cranial irradiation; POTRT, postoperative thoracic radiotherapy; pTNM, pathological tumor-node-metastasis; VATS, video-assisted thoracoscopic surgery.
